# Wearable Motion Sensors in Older Adults: On the Cutting Edge of Health and Mobility Research

**DOI:** 10.3390/s22030973

**Published:** 2022-01-27

**Authors:** Carl-Philipp Jansen, Katharina Gordt-Oesterwind, Michael Schwenk

**Affiliations:** 1 Department of Clinical Gerontology and Geriatric Rehabilitation, Robert Bosch Hospital, 70376 Stuttgart, Germany; 2 Institute of Sports and Sports Sciences, Heidelberg University, 69120 Heidelberg, Germany; gordt@issw.uni-heidelberg.de; 3 Network Aging Research (NAR), Heidelberg University, 69115 Heidelberg, Germany; schwenk@nar.uni-heidelberg.de; 4 Human Performance Research Centre, Department of Sport Science, University of Konstanz, 78457 Konstanz, Germany

Wearable motion sensors have been gaining ground for quite some time now; a large proportion of research projects in the field of physical activity, health, and mobility are being carried out using an electronic form of outcome assessment, and there are good reasons for this. Without discrediting other forms of assessment (they all have their raison d’être), patient-reported outcomes, as well as objective and subjective clinical outcome assessments, can be enhanced, or their weaknesses addressed, by wearable sensors. Among the most familiar weaknesses are recall/response bias, burdensome execution of test procedures, and psychometric deficiencies [1,2]. Especially in older populations, the reliability and validity of these assessments are further jeopardized by underlying health conditions related to the cognition and function of the tested person [3].

Current opportunities to evaluate health before age-related decline in function and health occurs are generally limited to a few checkups per year. Precision health through preventive measures and early detection cannot be realized by waiting until the next doctor’s, visit but will rely on integrating sensor-based health monitoring into everyday life. Unsurprisingly, wearable sensors have seen increasing popularity in research and the consumer landscape in general.

In particular, wearable motion sensors offer great potential for health-promoting interventions in older persons, geriatric rehabilitation, patient care, and research. Continuous health monitoring and integrated diagnostic devices—worn on the body—can help to identify and prevent early manifestations of age-related functional decline and disease.

Against this background, we ran this Special Issue in 2019, with the aim to cover some ground on the various fields of research with wearable sensors in the heterogeneous population of older persons; it focuses on new approaches in the area of wearable motion sensor technology application in older adults, and includes a wide range of wearable sensors and various kinds of health-related applications. Of all of the manuscripts received, 17 articles were published in this Special Issue, illustrating the use of wearable devices to assess physical capacity and function; to evaluate rehabilitation measures; to inform telemedicine; to investigate the validity, applicability, and clinical value of devices and algorithms; and to compare their results with those of self-report data. We further included three review articles looking into behavioural activity rhythms and the assessment of balance, as well as the diagnosis of respective changes. Not all of the articles reported on work that was actually carried out with older persons, but those that did contributed to the development of devices or algorithms intended for this population. To further acknowledge and highlight the authors’ work, we want to give a brief overview of the publications, listed chronologically by the publication date.

The first article by Alice Coni and colleagues [4] took up the challenge of developing a conceptual model based on a battery of smartphone-instrumented functional tests. By use of exploratory factor analysis, they were able to boil down the large number of available measures to a few domains that are able to advance quality, objectivity, and granularity of previously “analog” data.

In the second article, Gomes et al. [5] addressed the often neglected but highly important issue of nutrition monitoring. More specifically, they proposed a new algorithm to recognize eating and drinking in everyday life based on a new dataset. The ultimate goal was to trigger timely reminders for older persons living in their own homes, which can be a powerful tool to avoid malnutrition.

Pinho and colleagues [6] conducted a systematic review of the current state of the art in the device-based assessment of postural balance using mobile devices; the strengths and weaknesses of available solutions were evaluated. They found only nine papers, with no clear evidence on the accuracy of the devices to substantiate their use to assess balance, highlighting the necessity for more rigorous investigations of larger samples.

The systematic review by Leirós-Rodriguez et al. [7] investigated the same area as Pinho et al., with a focus on accelerometry to evaluate balance and balance alterations in older persons. Based on data from 19 papers, the authors concluded that there is still room for improvement, but highlight that valid and reliable measurements in laboratory settings are possible. Clinical use, however, is hampered by the potpourri of devices and uncertainties on the side of the end users.

Fillekes et al. [8] looked into GPS-based mobility assessment. They investigated the agreement between GPS-derived and self-report time out of the home and activity locations. They further investigated how agreement is altered by different spatial and temporal thresholds as well as the type and duration of the activities. While finding high agreement between self-reports and GPS reports for time out of the home, caution is needed when using more complex parameters such as activity locations.

Teufl et al. [9] addressed the issue of patients after total hip arthroplasty suffering from limited mobility due to musculoskeletal restrictions. The authors combined 3D gait kinematics, spatio–temporal parameters, and joint angles measured with inertial sensors (IMUs) in order to detect these impairments. They showed satisfying accuracy in measuring the kinematics using the IMU in subjects with different physiology. Further, using machine-learning approaches, they were able to differentiate between impaired and non-impaired gait in the standardized environment of the lab.

While using wearable sensors, researchers and clinicians are faced with the challenge of discriminating between standing, sedentary activity, and dynamic activities. To address this, Bijnens et al. [10] aimed to develop an adjustable algorithm for physical activity classification in an older population. While the classification of standing and sedentary phases showed acceptable results, the classification of dynamic activities still has some limitations.

Klenk and colleagues [11] carried out a prospective study on the psychometric properties of physical activity parameters to evaluate geriatric rehabilitation, and to link those parameters to clinical factors at admission. They found that sensor-based walking duration, walking bout duration, and the number of sit-to-stand transfers improved during rehabilitation, and that walking-duration progression was associated with Barthel Index scores, showing the clinical value of sensor-based assessment during in-patient rehabilitation as well as its relevance for the monitoring of physical activity progression.

Physical activity varies throughout the day, not only due to sleeping and waking phases. Neikrug et al. [12] developed a new approach to represent behavioral activity rhythms. This graphical presentation gives a more detailed insight into older adults’ daily routines and enables, for example, the tailoring of interventions very specifically to the behavioral rhythms of each person.

The study by Jung et al. [13] addresses the issue of fall-related injuries, especially hip fractures. They developed a fall detection algorithm for wearable airbags to minimize the impact of falls on the hips. Using IMU data, the algorithm could achieve high sensitivity, specificity, and accuracy in highly dynamic motions such as rapidly sitting down on, and getting up from, a chair, or jogging. However, authors underlined that further work is needed to use this algorithm for wearable airbags in real-life conditions.

The study by Trumpf et al. [14] compared habitual physical activity and sedentariness in older community-dwellers using a questionnaire and two different body-worn sensors: a wrist-worn actigraph and a hybrid motion sensor worn at the lower back. Physical activity analysis of the actigraph led to an overestimation compared to the hybrid motion sensor and the questionnaire, while sedentariness was underestimated by the actigraph. These findings underline the necessity to carefully select the sensor type based on the population and question of interest.

Not only can the classic gait parameters be measured with wearable sensors, but arm swing is also an important factor, especially in different patient populations such as those with Parkinson’s disease. Rincón et al. [15] aimed to use a low-cost wearable sensod system to detect early changes in arm swing as an assistive diagnostic tool. The developed algorithms were able to differentiate between healthy subjects and patients with Parkinson’s Disease by analysing the captured data of wristbands on each arm, which measured linear and angular acceleration. These findings enable clinicians to use this easy, low-cost system as an assistive diagnostic tool. 

Bortone et al. [16] looked into the discrepancy between self-report and objectively measured physical activity. In a sub-cohort of participants aged 65 years or older, the energy expenditure level measured using an actigraph on the dominant wrist was able to predict physical activity assessed by a structured interview questionnaire. However, it has to be considered that the sample was rather inactive, and larger measurement errors may occur in fitter populations.

Werner et al. [17] investigated the psychometric properties of a body-fixed sensor for the assessment of gait parameters in acute geriatric inpatients during rollator-assisted walking. While the parameters related to gait volume and pace in general showed good to excellent psychometric properties, the parameters related to symmetry and variability were inferior. From a practical perspective, they showed the high value of the sensor system to measure the most widely used gait parameters such as gait speed, step length and time, cadence, and walk ratio in older patients using rollators; however, they also highlight that caution needs to be in place when evaluating further parameters such as gait variability.

The main objective of the study by Kantoch et al. [18] was to investigate features and applications of telemedical services that are being delivered by wearable medical devices for the Polish older adults. A large proportion of study participants of all ages and socio–demographic groups was unaware of the telemedical services and opportunities offered to older adults. Retrospectively speaking, however, this study was conducted before the COVID-19 pandemic, which may have influenced the awareness and knowledge about telemedical services in general.

The identification of in-hospital fall risk factors of geriatric patients with dementia during hospitalization was the aim of Hauer et al. [19]. Besides “classic” geriatric assessments including neuropsychiatric and motor function, instrumented physical capacity assessments were carried out, showing that sensor-based balance parameters and specific cognitive sub-performances can especially be used to identify persons with dementia at risk of in-hospital falls.

Lastly, Schootemeijer et al. [20] investigated the association between daily-life gait quality parameters and fall risk in older persons. Sensors were attached using an elastic belt around the hip for one week; data showed that the logarithmic rate of divergence in the antero–posterior and vertical direction are especially associated with fall risk, being an adequate assessment of physiologic fall risk.

The articles included in our Special Issue clearly demonstrate the potential of wearable sensors for analysing the age-related parameters of physical capacity and physical activity. On the same note, the studies show that several approaches are still under development, and challenges lie ahead in validating new health-monitoring technologies and in optimizing data analytics to extract actionable conclusions from data obtained by wearable sensors. The advancement in various fields such as biomedical engineering and technology, artificial intelligence, and material science may overcome several of these challenges. Progressive reductions in costs, size, and energy requirements of wearable sensors, along with improved activity recognition algorithms, suggest that wearable systems may become ubiquitous tools. For instance, wearables have evolved to being capable of running artificial intelligence algorithms in real-time at the point of sensing, which allows researchers to gain analytical insights directly from measurement data [21]. User-friendliness to maintain long-term user engagement may be boosted by tattoo sensors and implanted sensors transmitting data wirelessly [22]. In precision health, data from wearables, implantables, and monitoring devices in the home would be aggregated to deepen the understanding of individuals’ health over the course of their lifespan.

Various sensors have been integrated into watches, wristbands, tattoos, belts, and smartphones, with the aim of monitoring, collecting, and transferring information about body movements, heart rate, and blood pressure to authorities such as health insurance companies, physicians, and consultants [23]. Apart from the challenge of data privacy and security, sooner or later, healthy aging and the prevention of age-related functional decline will most likely rely on technology-based products. This will not only improve the medical care of individuals and patients, but will also enable them to become actors on their own behalf based on valid and reliable sensor data.

As a final conclusion, wearable sensor approaches represent an ultimate option to foster healthy aging, and will most likely lead to massive progress the modern life for decades to come.

## References

[1] Del Din S., Godfrey A., Mazza C., Lord S., Rochester L. (2016). Free-Living Monitoring of Parkinson’s Disease: Lessons from the Field. Mov. Disord..

[2] Allet L., Knols R.H., Shirato K., de Bruin E.D. (2010). Wearable Systems for Monitoring Mobility-Related Activities in Chronic Disease: A Systematic Review. Sensors.

[3] Hauer K., Lord S.R., Lindemann U., Lamb S.E., Aminian K., Schwenk M. (2011). Assessment of Physical Activity in Older People with and without Cognitive Impairment. J. Aging Phys. Act..

[4] Coni A., Mellone S., Colpo M., Guralnik J.M., Patel K.V., Bandinelli S., Chiari L. (2019). An Exploratory Factor Analysis of Sensor-Based Physical Capability Assessment. Sensors.

[5] Gomes D., Mendes-Moreira J., Sousa I., Silva J. (2019). Eating and Drinking Recognition in Free-Living Conditions for Triggering Smart Reminders. Sensors.

[6] Pinho A.S., Salazar A.P., Hennig E.M., Spessato B.C., Domingo A., Pagnussat A.S. (2019). Can We Rely on Mobile Devices and Other Gadgets to Assess the Postural Balance of Healthy Individuals? A Systematic Review. Sensors.

[7] Leirós-Rodríguez R., García-Soidán J.L., Romo-Pérez V. (2019). Analyzing the Use of Accelerometers as a Method of Early Diagnosis of Alterations in Balance in Elderly People: A Systematic Review. Sensors.

[8] Fillekes M.P., Kim E.-K., Trumpf R., Zijlstra W., Giannouli E., Weibel R. (2019). Assessing Older Adults’ Daily Mobility: A Comparison of GPS-Derived and Self-Reported Mobility Indicators. Sensors.

[9] Teufl W., Taetz B., Miezal M., Lorenz M., Pietschmann J., Jöllenbeck T., Fröhlich M., Bleser G. (2019). Towards an Inertial Sensor-Based Wearable Feedback System for Patients after Total Hip Arthroplasty: Validity and Applicability for Gait Classification with Gait Kinematics-Based Features. Sensors.

[10] Bijnens W., Aarts J., Stevens A., Ummels D., Meijer K. (2019). Optimization and Validation of an Adjustable Activity Classification Algorithm for Assessment of Physical Behavior in Elderly. Sensors.

[11] Klenk J., Wekenmann S., Schwickert L., Lindemann U., Becker C., Rapp K. (2019). Change of Objectively-Measured Physical Activity during Geriatric Rehabilitation. Sensors.

[12] Neikrug A.B., Chen I.Y., Palmer J.R., McCurry S.M., Von Korff M., Perlis M., Vitiello M.V. (2020). Characterizing Behavioral Activity Rhythms in Older Adults Using Actigraphy. Sensors.

[13] Jung H., Koo B., Kim J., Kim T., Nam Y., Kim Y. (2020). Enhanced Algorithm for the Detection of Preimpact Fall for Wearable Airbags. Sensors.

[14] Trumpf R., Zijlstra W., Haussermann P., Fleiner T. (2020). Quantifying Habitual Physical Activity and Sedentariness in Older Adults—Different Outcomes of Two Simultaneously Body-Worn Motion Sensor Approaches and a Self-Estimation. Sensors.

[15] Rincón D., Valderrama J., González M.C., Muñoz B., Orozco J., Montilla L., Castaño Y., Navarro A. (2020). Wristbands Containing Accelerometers for Objective Arm Swing Analysis in Patients with Parkinson’s Disease. Sensors.

[16] Bortone I., Castellana F., Lampignano L., Zupo R., Moretti B., Giannelli G., Panza F., Sardone R. (2020). Activity Energy Expenditure Predicts Clinical Average Levels of Physical Activity in Older Population: Results from Salus in Apulia Study. Sensors.

[17] Werner C., Heldmann P., Hummel S., Bauknecht L., Bauer J.M., Hauer K. (2020). Concurrent Validity, Test-Retest Reliability, and Sensitivity to Change of a Single Body-Fixed Sensor for Gait Analysis during Rollator-Assisted Walking in Acute Geriatric Patients. Sensors.

[18] Kańtoch E., Kańtoch A. (2020). What Features and Functions Are Desired in Telemedical Services Targeted at Polish Older Adults Delivered by Wearable Medical Devices?—Pre-COVID-19 Flashback. Sensors.

[19] Hauer K., Dutzi I., Gordt K., Schwenk M. (2020). Specific Motor and Cognitive Performances Predict Falls during Ward-Based Geriatric Rehabilitation in Patients with Dementia. Sensors.

[20] Schootemeijer S., Weijer R.H.A., Hoozemans M.J.M., van Schooten K.S., Delbaere K., Pijnappels M. (2020). Association between Daily-Life Gait Quality Characteristics and Physiological Fall Risk in Older People. Sensors.

[21] Mirmomeni M., Fazio T., von Cavallar S., Harrer S., Sazonov E. (2020). Chapter 12-From wearables to THINK-ables: Artificial intelligence-enabled sensors for health monitoring. Wearable Sensors.

[22] Gambhir S.S., Ge T.J., Vermesh O., Spitler R., Gold G.E. (2021). Continuous Health Monitoring: An Opportunity for Precision Health. Sci. Transl. Med..

[23] Kang M., Park E., Cho B.H., Lee K.S. (2018). Recent Patient Health Monitoring Platforms Incorporating Internet of Things-Enabled Smart Devices. Int. Neurourol. J..

